# Outcome of Surgical Fixation for Midfoot Charcot Neuroarthropathy - A Systematic Review

**DOI:** 10.5704/MOJ.2303.004

**Published:** 2023-03

**Authors:** SL Ong, MY Bajuri, N Mazli

**Affiliations:** Department of Orthopaedics and Traumatology, Universiti Kebangsaan Malaysia, Kuala Lumpur, Malaysia

**Keywords:** Charcot, neuroarthropathy, midfoot, surgical reconstruction

## Abstract

**Introduction:**

Charcot arthropathy is a condition which is progressive, non-infectious, destructive and debilitating that commonly affect foot and ankle. This systematic review is to evaluate the occurrence of common outcomes associated with each intervention of Charcot neuroarthropathy in midfoot.

**Materials and methods:**

A systematic review on literatures that were published from Jan 2010 to Jan 2020 were collected, reviewed and selected regarding the surgical treatment procedures of Charcot neuroarthropathy in midfoot.

**Results:**

The initial search yielded 231 reports and after exclusion, nine out of the total studies were included in the outcome analysis for review. These were studies that included data concerning surgical reconstruction of Charcot arthropathy in the midfoot.

**Conclusion:**

It is suggested that soft tissue preparation and usage of combination of implants thus reduce the risk of infection as well as increase rigidity of construct, respectively. These factors will aid to improve outcome of midfoot Charcot arthropathy reconstruction.

## Introduction

Charcot arthropathy is a condition which is progressive, non-infectious, destructive and debilitating that commonly affect foot and ankle. It is a complicated yet poorly been described condition that posed a great challenge to the treating surgeon. Although diabetes is the primary driver of Charcot neuroarthropathy, other neuropathic morbidities such syphilis, leprosy, spinal cord injury, syringomyelia, toxic exposure, spinal cord injury, rheumatoid arthritis, poliomyelitis, multiple sclerosis, cellulitis, congenital neuropathy, osteomyelitis, synovitis, and alcohol abuse have also been associated with the disease^1,2^. Exact pathophysiology of Charcot arthropathy is not fully understood. Neurotraumatic theory (in 1917 by Elosser then Johnson in 1967) and neurovascular theory (by Brower in 1981) were proposed pathogenesis of development of Charcot arthropathy^1^.

There are various anatomical classifications of Charcot neuroarthropathy. Both Sander/Frykberg and Brodsky classification include the whole foot, whereas Schon specifically classified deformity over midfoot only^1^. Another classification by Eichenholtz explained three stages of disease progression based on clinical signs and radiographic findings^1^. Charcot arthropathy commonly affects the midfoot with incidence of 60% followed by ankle and hindfoot^1^. It is always difficult to recognise Charcot neuroarthropathy due to similar initial presentation with other inflammatory or infection conditions such as cellulitis, osteomyelitis, arthritis and even ligamentous injury of the ankle. These conditions usually presented with redness and swelling around affected side. As the disease progresses, the midfoot collapse, described as a “rocker-bottom” foot which increases peak plantar pressure and thus lead to recurrent ulcer. This may predispose patient to a devastating outcome such as amputation.

The goal of management of Charcot neuroarthropathy is to produce a stable, shoe-able, painless and plantigrade feet that are free from ulceration, hence reducing risk of ascending infection, osteomyelitis, and amputation. The initial preferred management on acute Charcot neuroarthropathy is immobilisation via total contact cast or special orthoses. Surgical reconstruction is indicated when infection, recurrent ulceration and unstable joint occurred. Several researchers tried to reveal the outcome of midfoot reconstruction in various methods either internal or external fixations.

However, there are no existing standard protocol for the surgical management of Charcot arthropathy due to the heterogeneity of both the disease entity and clinical presentation. Therefore, we conduct a systematic review on literatures that were published from Jan 2010 to Jan 2020 regarding the surgical treatment procedures of Charcot neuroarthropathy in midfoot. Intramedullary fixation which is medial column bolt fusion, external fixation and plating were the main operative interventions explored in this study. We also plan to evaluate the occurrence of common outcomes associated with each intervention. We expect to provide the latest advancements in the surgical treatment of Charcot neuroarthropathy and reveal the complications involved with each procedure, thus preventing unwanted outcomes upon reconstructing the midfoot deformity which was caused by Charcot neuroarthropathy.

## Materials and Methods

**Fig. 1:**
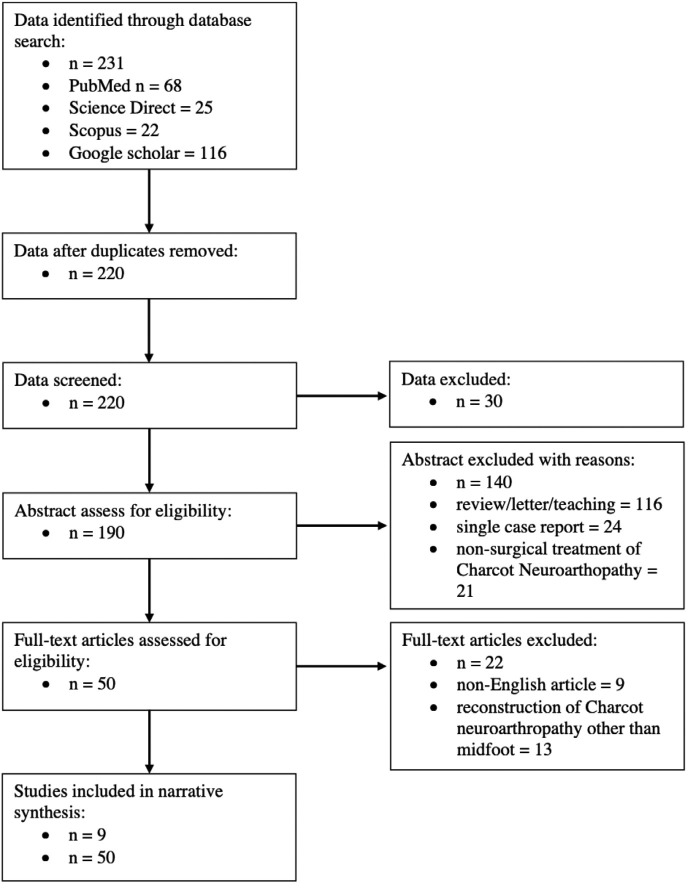
Preferred reporting items for systematic reviews and meta-analyses diagram.

This systematic review uses the preferred reporting items for systematic reviews and meta-analysis (PRISMA) checklist (Fig. 1). Data were collected via Medline (Ovid, PubMed), Science Direct, Scopus and Google scholar by using terms: Charcot neuroarthropathy, neuro-osteoarthropathy, osteoarthropathy, neurogenic arthropathy, foot, midfoot, surgical offloading, diabetic reconstruction, external fixation, internal fixation. This study includes all English published original paper from Jan 2010 till Jan 2020, comprises of all human or, case control studies, randomised cross over studies, randomised controlled trials, randomised cross pilot studies, pre-post design studies, surgical management at each stage of Charcot arthropathy, article which long term outcome post intervention on Charcot neuroarthropathy is mentioned. We excluded paper that are not published in English, case report, animal studies, letters to editor and review article. The terms Charcot arthropathy, neuroarthropathy, neuropathic arthropathy, and neuropathic osteoarthropathy were used interchangeably for the purposes of this study.

The search was started since May 2019. All articles were screened independently by primary author and second author in three phases: title, abstract and full-text screening. The number of articles on which all our reviewers agreed in terms of inclusion and exclusion was divided by the total number of double screened papers to determine inter-observer agreement. Discrepancies among both primary and second author was resolved by consensus. For full text assessment and data extraction, we included all articles that are eligible following our consensus.

We gathered information on the demographics of the patients included (Table I), method of reconstruction, infection, union, amputation, and hardware complication. We also extracted information on method of application of technique. Data were extracted from all included articles by the first author. The second author independently validated the completed data extraction sheets against the articles.

**Table I:** Patient demographic

Investigator	Evidence grading (level)	Study design and time frame for data collection	Sample size and CN classification	Participant’s characteristics	
Cullen et al^6^ (2013, USA)	IV	Case series (March 2010 to August 2011)	4 Not specified classification	3 males 1 female	47 to 70 years old (average: 57.3)
Eschler et al ^8^ (2014, Germany)	IV	Retrospective case series (May 2009 to March 2012)	7 Eichenholtz I : 2 II : 5	7 males 0 female	47 to 68 years old (average: 56.3)
Ford et al^5^ (2019, USA)	IV	Retrospective case series (January 2010 to July 2016)	25 Eichenholtz 0 : 1 I : 4 II : 10 III : 10	13 males 12 females	Age years mean=58
Garchar et al^7^ (2013, USA)	III	Retrospective case series (April 1999 to July 2004)	24 all patient either late stage II or stage III	14 males 10 females	42 to 74 years old (average: 58.8)
Lamm et al^3^ (2010, USA)	IV	Retrospective case series (November 2003 to July 2006)	8 Eichenholtz I : 1 II : 6 III : 4	3 females 5 males	41 to 79 years old (average: 61)
Matsumoto et al^7^ (2015, USA)	IV	case series (December 2009 to May 2013)	10 Unable to summarise classification from article	4 males 6 females	35 to 64 years old (average: 52.2)
Mehlhorn et al^10^ (2016, Germany)	IV	Retrospective case series (April 2011 to September 2015)	14 all patient either late stage II or stage III	9 males 5 females	Age years mean=59
Richter et al^9^ (2015, Germany)	IV	Multicenter case series (November 2003 to July 2006)	47 Eichenholtz (with 10 not assess) I : 26 II : 6 III : 6	28 males 19 females	35 to 78 years old (average: 60.1)
Wiewiorski et al^11^ (2013, Switzerland)	IV	Retrospective case series (October 2007 to July 2010)	8 Sammarco (with 1 not assess) 1 : 3 2 : 0 3 : 3 5 : 1	6 males 2 females	46 to 80 years old (average: 63)

## Results

The initial search yielded 231 reports and after the duplicates were lifted, we have 220 papers. Thirty of it were excluded during title screening and 140 out of 190 abstracts were excluded as those were review article, single case report and non-surgical treatment on Charcot neuroarthropathy. The remaining 50 full texts were read, and 22 were removed from the study because they had included patients who had undergone Charcot neuroarthropathy surgery in location other than midfoot and did not have separate results for the Charcot patients receiving surgery. Nine out of the 18 studies were included in the outcome analysis for review. These were studies that included data concerning surgical reconstruction of Charcot arthropathy in the midfoot.

Most of the studies were retrospective studies with level of evidence of IV (8 out of 9) and remaining study carry level of evidence of III. The most cited procedures were intramedullary medial column bolt fusion and multilevel external fixation. Studies were identified regarding other procedures such as Achilles tendon lengthening, combined internal and external fixation. The table below lists the data regarding the outcome of these studies (Table II).

**Table II:** Outcome of these studies

Investigator	Surgical Procedure	Additional procedure	Surgeries analysed	Infection	Hardware complication	Amputation	Union
Cullen et al^6^ (2013, USA)	MFB	TA lengthening and external fixation	4	1	1	0	3
Eschler et al^8^ (2014, Germany)	MFB	Screw or plate	7	5	2	2	5
Ford et al5 (2019, USA)	MFB	Excision of ulcer and osteotomies	25	6	1	4	21
Garchar et al^4^ (2013, USA)	Plantar plate	2 cortical screw and TA lengthening	25	4	0	0	24
Lamm et al^3^ (2010, USA)	External fixation	screw	11	11	9	0	11
Matsumoto et al^7^ (2015, USA)	Multiaxial correction Fixator	Plate or screw, (TA lengthening in 6 cases)	11	1	0	0	11
Mehlhorn et al^10^ (2016, Germany)	MFB	TA lengthening	14	7	9	3	8
Richter et al^9^ (2015, Germany)	MFB	Plate, screw, wire, TA and gastrocnemius lengthening	48	10	4	5	N/A
Wiewiorski et al^11^ (2013, Switzerland)	MFB	Plate, screw	8	1	3	0	8

Abbreviations - MFB: Midfoot Fusion Bolt, TA: Tendon Achilles, N/A: Not Available

Five studies were conducted in the United States^3-7^, three studies in Germany^8-10^ and one in Switzerland^11^. Data were collected in these studies range between 20 months to 6 years. Overall, 153 foot (148 patients) were included in these studies with 89 male and 59 females with age ranges from 35 to 78 years old. The number of patients in each study ranges from 4 to 47. Most of the neuropathy associated with Charcot among these studies is diabetes mellitus, however one study does not provide cause of underlying Charcot neuroarthropathy among subjects. Four studies include reconstruction of Charcot in acute phase (Eichenholtz stage I)^3,5,8,11^. Thirty-three patients in the acute phase were treated with internal fixation (midfoot bolt and plate or screw) in three retrospective series and one retrospective series using method with external fixation as first stage of reconstruction followed by internal fixation. The remaining studies that discussed surgical treatment of Charcot neuroarthropathy focused on reconstruction in the later stages after coalescence had occurred and/or failed conservative management (Eichenholtz stage II and III). Six out of nine studies applied midfoot bolt as the mode of reconstruction in Charcot neuroarthropathy^5,6,8-11^, two applied external devices^3,7^ and the last study used the plate as a tool for reconstruction^4^.

## Discussion

Charcot neuroarthropathy patients experience a higher rate of morbidity and a lower quality of life. Charcot arthropathy consistently become a challenge to us even with most experienced foot ankle surgeon^12,13^. The main aim of reconstruction in Charcot patients is to achieve a stable, shoe-able, plantigrade and painless foot that is free from ulceration. Surgical intervention of Charcot neuroarthropathy is challenging due to the complexity of the disease entity^13^. Various surgical methods for internal and external fixation have been reported for use in reconstruction of Charcot arthropathy however they have been associated with high rates of complications. The notion of “super-construct” which was created by Sommarco aim to achieve a stable reconstruction of Charcot arthropathy. The stable construct may reduce probability of complication which can jeopardise patient quality of life. It is defined by four factors:

(1) fusion extended beyond the injury zone including joints that were not affected, (2) shortening the affected limb with bone resection to allow for adequate reduction without unwanted tension on the soft tissue envelope, (3) the strongest device that the soft tissue envelope can tolerate is used, and (4) the devices are applied in a position that its mechanical function is maximised. In midfoot, super-construction can be achieved by plantar plate, axial screw fixation and locking plate. However, in the data analysed, there were no direct comparisons about the methods of fixation. The most described intervention among studies collected were intramedullary medial column bolt fusion and multilevel external fixation.

Union post reconstruction and a stable ankle aid to enhance the quality of life of Charcot arthropathy patients. Most studies suggest combination of fixations to increase the stability of construct and improve union rate^3-6,8,14^. The additional fixations act as supplement to improve outcome after reconstruction of midfoot Charcot. Lamm *et al* used circular external fixator for gradual distraction in first stage of treatment to prevent neurovascular compromise followed by internal fixation in second stage for reconstruction^3^. Gradual traction of the affected limb also aids in reducing soft tissue tension upon correction of deformity^3^. In most of the studies (five out of nine) combination of Achilles tendon lengthening upon reconstruction aids in reducing soft tissue tension and ease for reduction upon realignment of Charcot joint^4,6,7,9,10^.

Single mode of implant is not recommended as a solitary midfoot bolt is insufficiently stable biomechanically. It acts as the centre of rotation which predispose the construct into rotational instability^7,9^. A supplementary lag screw with a larger diameter will reduce the bending stress in a solitary midfoot bolt and hence prevent hardware complication^8,10^. Study by Eschler *et al* with solitary midfoot bolt shows a significant incidence of migration (implant loosening) which required removal post-operatively^8^. High union rate in 24 out of 25 feet (96%) was noted by Garchar *et al* when a plantar plate was accompanied with two multi-axial screws^4^. A more favourable outcome can be demonstrated by increasing the number of beams in both axial and sagittal plane of midfoot as it diverts the concentration of bending stress^5,9,15^.

There is a dilemma by choosing cannulated versus solid midfoot bolt by weighting between mechanical strength and risk of infection. Cannulated screw often chosen for reconstruction as there is less soft tissue dissection and small incision upon application of the device^8^. It has higher risk of breakage (39%) when compared to a solid core screw upon weight bearing in Charcot patient post reconstruction^7,16^. There is a higher technical advantage in midfoot bolt due to its headless design with solid core. However, there is a drawback due to axial migration when it is utilised solitarily for medial column stabilisation without resection of joint surface^11^.

Two out of nine studies utilise external fixator in midfoot Charcot reconstruction prior to intramedullary fixation with screw and midfoot fusion bolt^3,6^. In both studies, external fixator aids in gradual distraction to regain osseous alignment prior to fusion via internal device and prevent neurovascular compromise^3,6^. Upon our review, one study used solitary method with multiaxial correction fixator in combination with plate in midfoot reconstruction^7^. Lamm *et al* reported 100% pin tract infection as complication of external fixator^3^. The author did not specify the factor that resulted in pin tract infection, but there were no records of non-union, deep infection and requirement for hospitalisation among the patients. However, there was no pin tract infection noted in study by Matsumoto and Parekh^7^. It is because less number of pins were used and the pins were inserted away from muscle and tendon that prevent soft tissue irritation^7^.

Charcot arthropathy is a non-infective disease but secondary infections are common, and it is a devastating condition for the treating surgeon. Eichenholtz explained three stages of disease progression based on clinical signs and radiographic finding. In stage 1, which is fragmentation stage, patient may present with a swollen, warmth and lax joint as well as an erythematous limb. Radiographically, affected limbs may have periarticular debris, fragmentation, and subluxed or dislocated joint with underlying osteopenic bone. It is followed by coalescence stage (II) in which redness, swelling and warmness been reduced. Periarticular debris will be absorbed, sclerosis and consolidation over large fragment can be seen in radiographs. Resolution as the final stage (III) in Charcot neuroarthropathy as per describe by Eichenholtz where the affected joint progress into a more stable position with no erythematous, swelling and warmness. However, the joint will be in a stable and fixed deformity. While radiologically, bone fragment that consolidate will have a smoother and round edge, joint space will be reduced, ankylosis maybe seen.

In previous studies, intervention was done at Eichenholtz stage II or III as there is higher risk of infection when it was done in earlier stage. However, there are five out of nine studies^3,5,8,9,11^ had done reconstruction in stage I, in which three of the studies record a 100% union rate^3,8,11^. The other two studies reported union rate of 98% and 84%, respectively^5,19^. Study by Garchar *et al* and by Mehlhorn *et al* showed union rate of 57% and 96%, respectively when reconstruction was done either in late stage II or stage III^4,10^. The remaining two studies had 100% union but did not specify the stages of Charcot upon reconstruction^6,7^.

Infection in Charcot arthropathy may be due to the loss of the protective mechanism of foot, altered plantar pressure, and impaired of healing due to underlying comorbidity such as long-standing uncontrolled diabetes. Pre-reconstruction infection such as osteomyelitis and ulceration were found to be a risk factor for recurrent infection post reconstruction compared to those without ulceration prior to intervention^5,8^. An insulin dependent diabetic Charcot patient with Haemoglobin A1C more than 7% has a higher risk of postoperative infection. Ford et al and Cullen et al also noted that external fixation had higher risk of pin infection in patient with deranged haemoglobin A1C level^5,6^.

Ulcer debridement and osteotomy of osteomyelitis bone during reconstruction helps in preparing soft tissue envelop for implant coverage and prevent recurrence of ulcer and deformity due to implant failure^3,6^. Gradual deformity correction via minimally invasive procedure by B.M Lamm warrant a favourable outcome where less soft tissue and neurovascular compromise that reduce infection^3^. All subjects in the study were able to achieve plantigrade foot without deep infection and not recurrence of ulcer. Seven out of nine studies disclosed pre-operative ulceration prior to internal fixation. But there were two studies which did not mention ulcer occurrence per-reconstruction^4-6,8-11^. Amputation rate among these studies range between 10.41% to 28%^5,8-11^. However, 100% limb salvage noted on Wiewiorski et al study where they utilised beam stabilisation of medial column and midfoot as mode of reconstruction^11^.

Limitation of the present study is that the level of evidence collected in management of midfoot Charcot arthropathy (mostly level IV) which were retrospective study and therapeutic study which included a small number of subjects. This might be due to diversity of cases by each author and making management of surgical reconstruction in each aspect unique. We hope that there are studies with longer follow-up, larger subject population and more comparisons among fixation method in future to provide a higher validity suggestion on surgical fixation on Charcot Neuroarthropathy reconstruction.

## Conclusions

In conclusion, most of the neuropathy associated with Charcot among these studies is diabetes mellitus. We suggest that soft tissue preparation is important and application of midfoot bolt that combined with locking plate or screws increase rigidity of construct, and hence achieve union. Ilizarov fixation or external fixator usage will help to reduce the risk of infection. Most of the cases that involved midfoot reconstruction in Charcot arthropathy will have Achilles tendon lengthening or gastrocnemius resection. Higher construct failure rate was noted when isolated implant was applied.
